# An Anatomical Variation of the Anconeus Epitrochlearis Muscle: A Case Report

**DOI:** 10.7759/cureus.88743

**Published:** 2025-07-25

**Authors:** Andrew T Casteel, Braden R McNees, Joe Iwanaga, R. Shane Tubbs, Steven M Hill

**Affiliations:** 1 Neurosurgery, Tulane University School of Medicine, New Orleans, USA; 2 Structural and Cellular Biology, Tulane University School of Medicine, New Orleans, USA

**Keywords:** anconeus epitrochlearis, anomaly, cubital tunnel syndrome, elbow, variant

## Abstract

The anconeus epitrochlearis muscle (AEM) is an anatomical variant found in various animal species and some humans, believed to be evolutionarily linked to climbing behavior. It typically spans from the medial epicondyle of the humerus to the olecranon and is innervated by the ulnar nerve. While most AEMs appear as a single-headed muscle, this case report describes a rare three-headed AEM identified during routine cadaveric dissection of an 82-year-old female. On the right side, the AEM showed a typical single-headed structure. On the left side, however, the muscle had three distinct heads, with the proximal head located deep to the ulnar nerve, an unusual configuration not previously documented. All measurements were made using microcalipers. Clinically, the AEM has been associated with cubital tunnel syndrome due to potential ulnar nerve compression. However, some studies suggest it might protect the ulnar nerve compared to the more rigid Osborne’s ligament. Variants of the AEM are rare, and to our knowledge, this case of a three-headed AEM is likely the first such report in the literature, contributing new anatomical knowledge with potential clinical relevance.

## Introduction

The anconeus epitrochlearis muscle (AEM) has been reported in numerous animal species, including amphibians, reptiles, mammals, and human ancestors [1]. However, as humans have evolved, the AEM, which is believed to aid in climbing, has become more frequently absent. Thus, since its first reported case by Gruber in 1866, it has been considered an anatomical variant [2]. It originates from the medial epicondyle of the humerus, inserts onto the olecranon, and is innervated by the ulnar nerve [1-3]. While most consider it an extension of the medial head of the triceps brachii, some have proposed that it derives from the flexor carpi ulnaris [3]. When present, the AEM contributes to the roof of the cubital tunnel [4]. It tightens during elbow flexion and relaxes during elbow extension [5].

Few variations of the AEM other than size have been reported in the literature. Herein, we present a seemingly very rare finding of the AEM in a cadaveric donor. Although Gruber could describe minute differences in the AEM, few have been reported [2]. The muscle is often seen as having a single fibrous rectangular head, but the case presented herein demonstrated a unique three-headed anatomy.

## Case presentation

**Figure 1:**
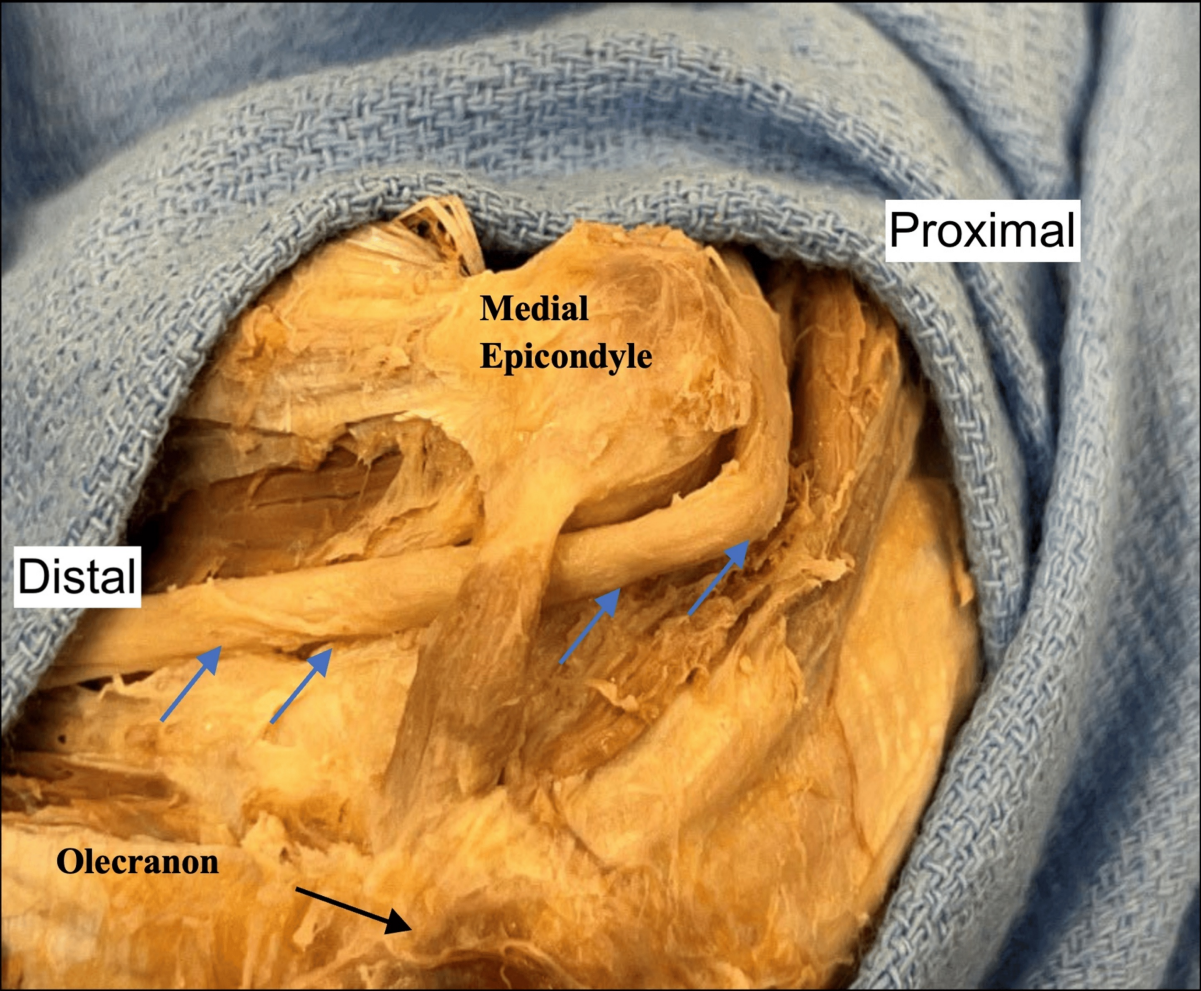
As presented on the right medial elbow of the female cadaver, the anconeus epitrochlearis demonstrates its typical anatomy and course from the medial epicondyle of the humerus to the olecranon (black arrow). The ulnar nerve (blue arrows) courses underneath the muscle through the cubital tunnel.

During the routine dissection of an adult female cadaver aged 82 years at death, an AEM was identified bilaterally. The right-sided muscle had a typical appearance, attaching from the medial epicondyle to the olecranon (Figure 1)

**Figure 2:**
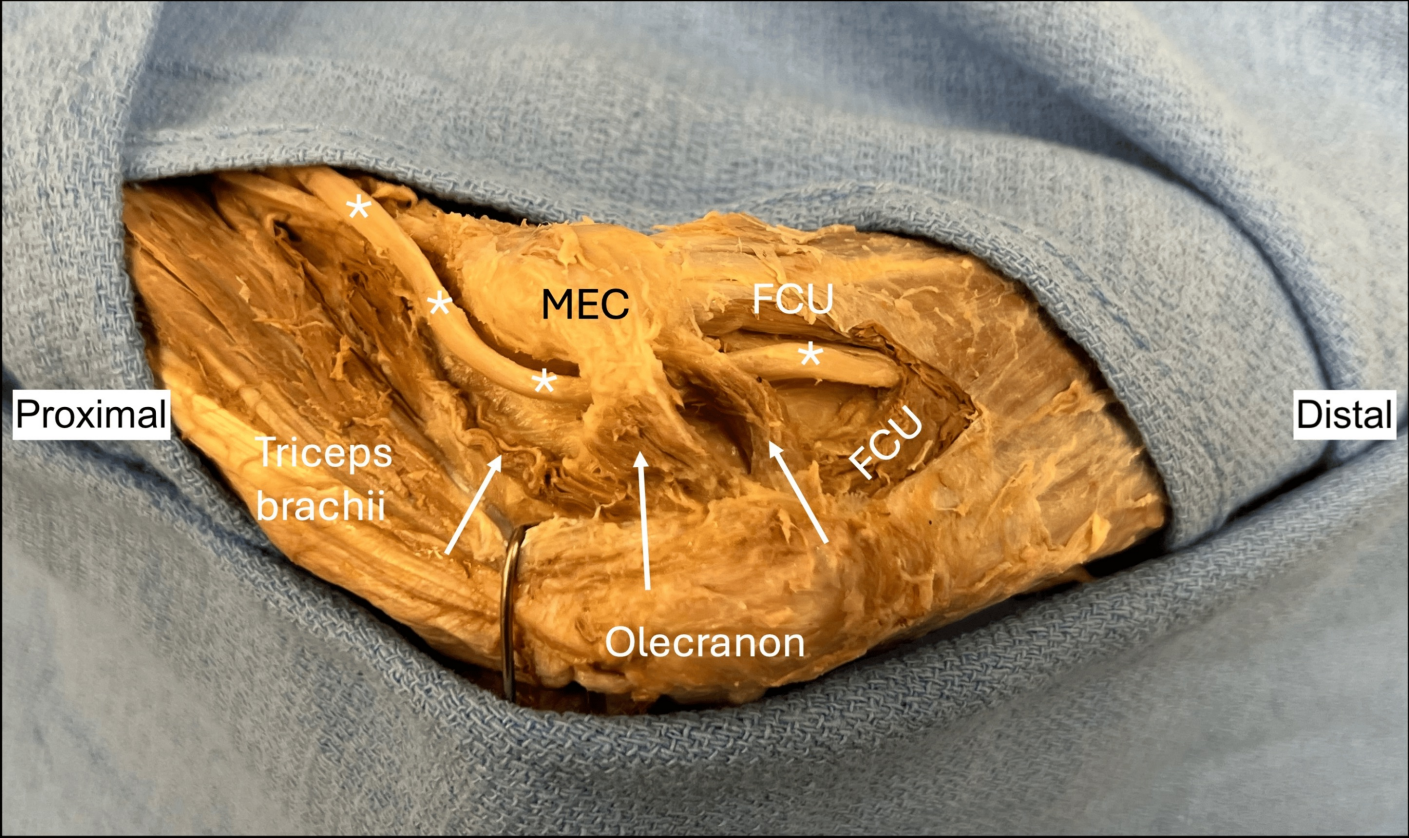
On the left medial elbow of the female cadaver, the anconeus epitrochlearis demonstrates its atypical anatomy but follows the typical course from the medial epicondyle of the humerus to the olecranon of the ulna. The ulnar nerve (*) courses underneath the muscle through the cubital tunnel. The three heads of the anconeus epitrochlearis (white arrows) can be seen. FCU: flexor carpi ulnaris; MEC: medial epicondyle

The right-sided muscle had a width of 8.5 mm and a length of 1.6 cm. The attachment onto the olecranon was primarily muscular, and the attachment onto the medial epicondyle was via a tendon of 6 mm in length. However, the AEM on the left side had three distinct heads (Figure 2).

The distal two heads traveled in parallel from the medial epicondyle to the olecranon, both crossing over the ulnar nerve and forming the roof of the cubital tunnel. The most proximal head arose from the olecranon in conjunction with the middle head, attached to the medial intermuscular septum and medial head of the triceps brachii, and traveled deep to the ulnar nerve. The middle head had a width and length of 7.5 mm and 1.4 cm, respectively. The distal head had a width of 4.5 and a length of 1.6 cm. The proximal head was 5.5 mm in width and 1.6 cm in length. No other anatomical variations were noted on either side. All measurements were made with microcalipers (Mitutoyo, Japan). 

## Discussion

The reported prevalence of the AEM varies widely in the literature. Since Gruber's initial report, it has been reported from 1% to 34%, irrespective of demographic factors [1,2]. Gruber researched the prevalence of the AEM in 100 cadavers. He found it in 26 males (57% bilateral prevalence) and eight females (50% bilateral prevalence) [2,6,7]. In Gruber’s paper, numerous animal species, including some primates, were consistently identified as having an AEM, suggesting an evolutionary link to humans [1,8].

The clinical relevance of the AEM is linked to cubital tunnel syndrome, but its role has been controversial since the preliminary study by Wilson et al. (2016), indicating that the muscle might have a protective role for the ulnar nerve at the elbow [4]. Cubital tunnel syndrome entails ulnar nerve entrapment within the cubital tunnel of the medial elbow. Symptoms include a loss of sensation to the entire fifth digit and the medial half of the fourth, atrophy of the fourth intermetacarpal space, and reduced strength during abduction of the fifth digit [9]. When the AEM contributes to cubital tunnel syndrome, this is believed to be secondary to muscle hypertrophy because of excessive use [4,7]. Compared to idiopathic cubital tunnel syndrome, ulnar nerve compression due to an AEM has been reported more often in the dominant arms of younger male patients [7]. Park et al. attributed this finding to increased activity levels in young men requiring excessive use of their upper limbs [7]. In 2016, a retrospective cohort study further investigated the relationship between the AEM and cubital tunnel syndrome [4]. The authors proposed that, owing to the more malleable nature of muscle in the AEM than the relatively rigid ligament in Osborne’s ligament, the AEM is preventative against cubital tunnel syndrome [4]. Additionally, they reported that the AEM was more prevalent in asymptomatic patients than in those undergoing surgical intervention for cubital tunnel syndrome [4].

Reports of variations of the AEM are scarce. Massrey et al. identified the left AEM in a fresh-frozen specimen from an 89-year-old female [6]. However, they noted in their case report that the AEM had proximal muscular extensions into the medial intermuscular septum anteriorly and posteriorly as it attached to the triceps brachii [6].

## Conclusions

The AEM should be considered in cases of ulnar nerve compression in the cubital tunnel. Moreover, given the present case report, anatomical variations of the AEM should also be considered. To our knowledge, a three-headed AEM has not been reported previously. Therefore, such a case is of archival value.
